# Supplementary material for: Last millennium hurricane activity linked to endogenous climate variability
Source: Nat Commun. 2024 Jan 27;15:816. doi: 10.1038/s41467-024-45112-6 (PMC10821936; doi:10.1038/s41467-024-45112-6)
Supplement: Supplementary file 1 — Supplementary Information [file 41467_2024_45112_MOESM1_ESM.pdf]

Supplementary Information for "Last Millennium Hurricane Activity linked to Endogenous Climate Variability"

Wenchang Yang*[1], Elizabeth Wallace[2], Gabriel A. Vecchi[1,3], Jeffrey P. Donnelly[4], Julien Emile-Geay[5], Gregory J. Hakim[6], Larry W. Horowitz[7], Richard M. Sullivan[2], Robert Tardif[6], Peter J. van Hengstum[8,9], Tyler S. Winkler[4,9],

[1]*Department of Geosciences, Princeton University*

[2]*Department of Earth and Ocean Sciences, Old Dominion University*

[3]*High Meadows Environmental Institute, Princeton University*

[4]*Department of Geology and Geophysics, Woods Hole Oceanographic Institution*

[5]*Department of Earth Sciences, University of Southern California*

[6]*Department of Atmospheric Sciences, University of Washington*

[7]*Geophysical Fluid Dynamics Laboratory, NOAA*

[8]*Department of Oceanography, Texas A&M University*

[9]*Department of Marine and Coastal Environmental Science, Texas A&M University at Galveston*

**email: wenchang@princeton.edu*

This PDF file includes:

- Supplementary Table S1
- Supplementary Figures S1 to S7

Supplementary Table

Supplementary Table S1: Location of paleohurricane sediment sites including their intensity threshold and modern return periods used for weights. Source data are provided as a Source Data file.

Location	Latitude	Longitude	Sensitivity	Return Period
Salt Pond, MA	41.54	-70.62	Cat 2	14.6
Mattapoissett marsh, MA	41.65	-70.78	Cat 2	15.74
Succotash marsh, RI	41.379	-71.521	Cat 2	15.62
Alder Island, NY	40.597	-73.58	Cat 2	17.21
Brigantine NJ	39.4	-74.36	Cat 2	13.59
Whale Beach NJ	39.18	-74.67	Cat 2	13.37
Segiune Pond, Staten Island, NY	40.52	-74.17	Cat 2	22.11
Singleton Swash, SC	33.76	-78.79	Cat 3	13.16
Mullet Pond, FL	29.925	-84.338	Cat 1	3.45
Spring Creek Pond, FL	30.098	-84.328	Cat 1	3.51
Shotgun Pond, FL	29.932	-84.355	Cat 1	3.44
Basin Bayou, FL	30.491	-86.245	Cat 3	13.86
Blackwood sinkhole, Abaco Island, Bahamas	26.79	-77.42	Cat 3	5.75
Thatchpoint Bluehole, Abaco Island	26.323	-77.293	Cat 2	3.89
South Andros Island, Bahamas	23.779	-77.719	Cat 3	7.04
Long Island, Bahamas	23.265	-75.118	Cat 2	4.91
Middle Caicos Island, TCI	21.7198	-71.8128	Cat 1	3.13
Lagune Playa Grande, Vieques, PR	18.089	-65.517	Cat 3	6.53
Cenote Muyil, Yucatan Peninsula	20.04	-87.06	Cat 3	11.17
Lighthouse Reef blue hole, Belize	17.315	-87.534	Cat 1	4.96

Supplementary Figures

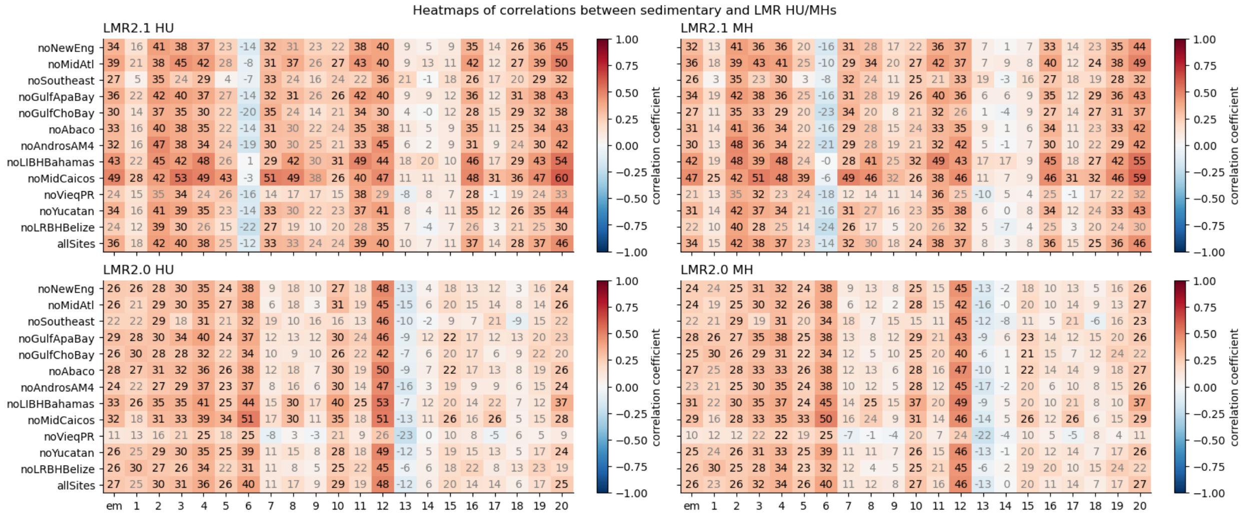

Supplementary Figure S1: Pearson correlation coefficients between sedimentary reconstructed hurricanes (HUs) along with its Jack-knife members and emulated HU (left column) and major hurricanes (MH, right column) using Last Millennium Reanalysis (LMR) 2.1 (top row) and LMR 2.0 (bottom row) sea surface temperatures (SSTs). The numbers are the coefficients multiplied by 100, for which black and gray colors indicate significant and non-significant values, respectively, under the one-side t-test at the 0.1 level using effective sample size. Source data are provided as a Source Data file.

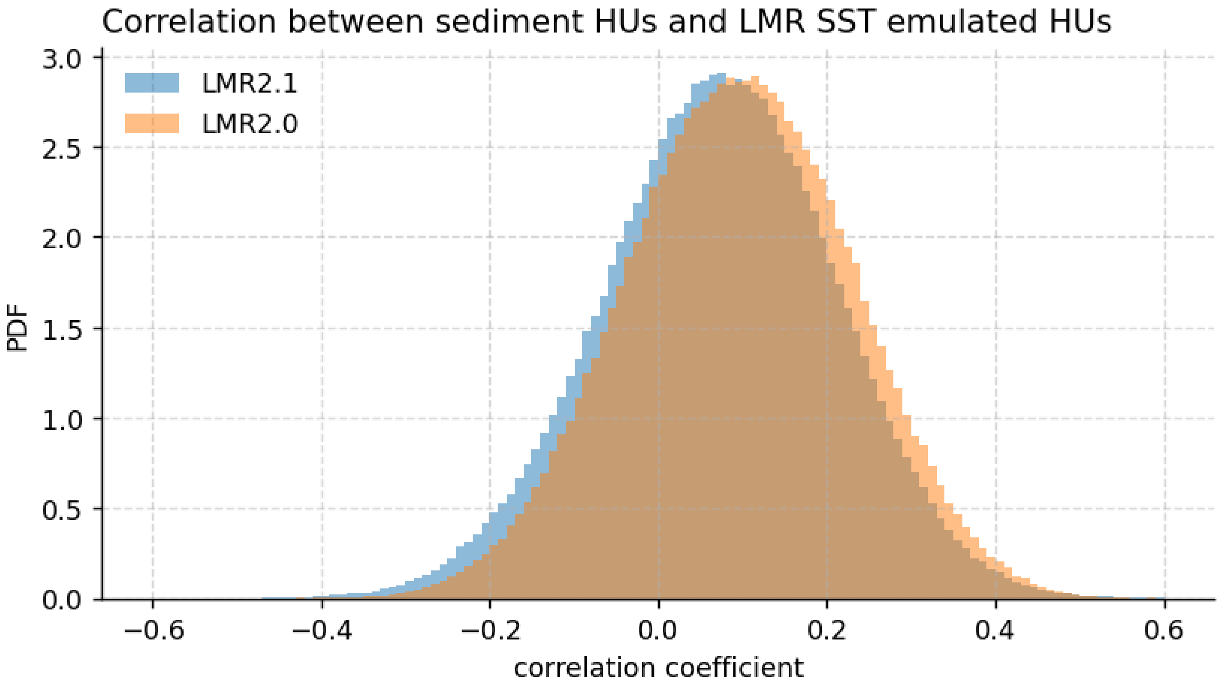

Supplementary Figure S2: Pearson correlation coefficients between all Monte Carlo members of sedimentary reconstructed hurricanes (HUs) and all ensemble members (also including the ensemble mean) of Last Millennium Reanalysis (LMR) sea surface temperature (SST) emulated HUs. Source data are provided as a Source Data file.

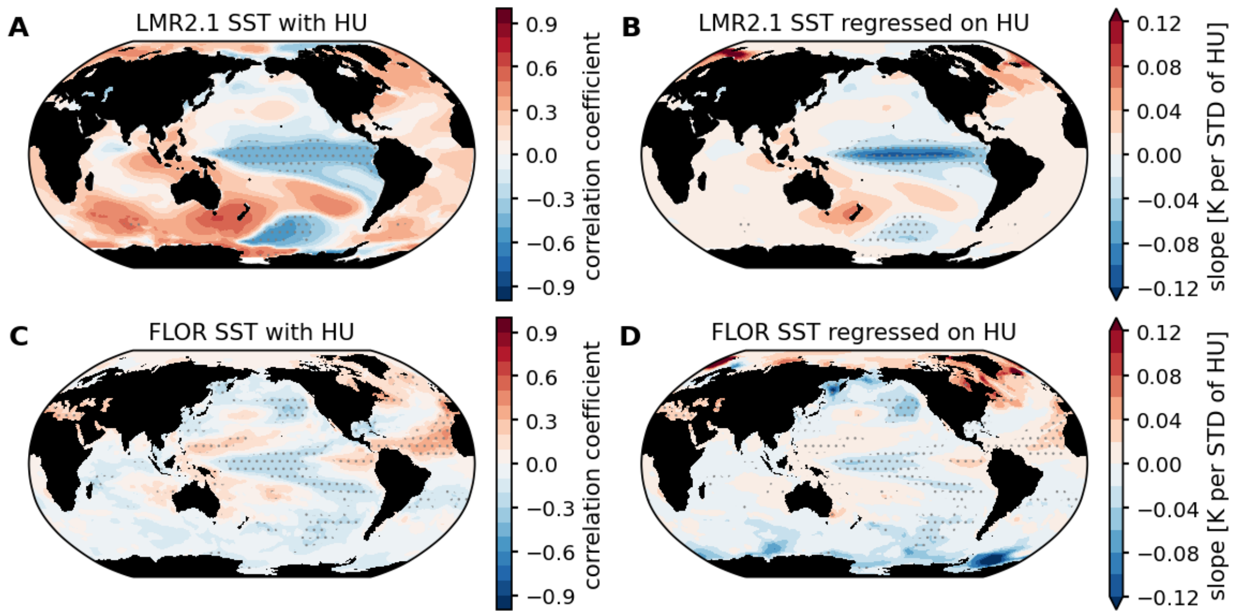

Supplementary Figure S3: Same as Figure 3 except that sea surface temperatures (SSTs) instead of relative SSTs are used. Source data are provided as a Source Data file.

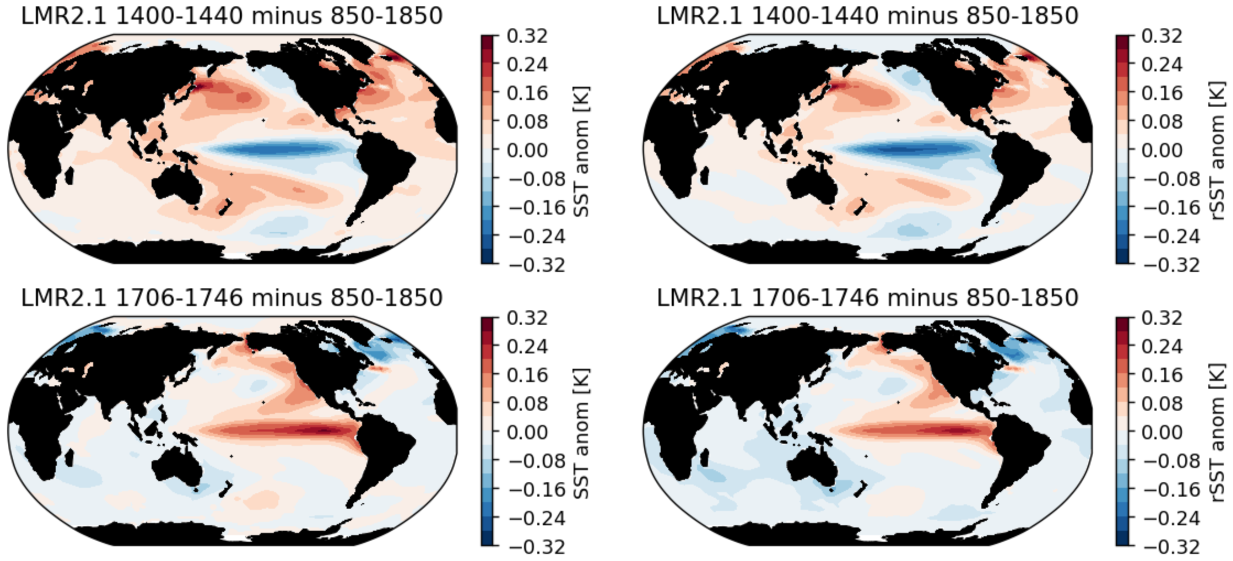

Supplementary Figure S4: Maps of Last Millennium Reanalysis (LMR) 2.1 sea surface temperature (SST) and relative SST anomalies over some historical periods. Source data are provided as a Source Data file.

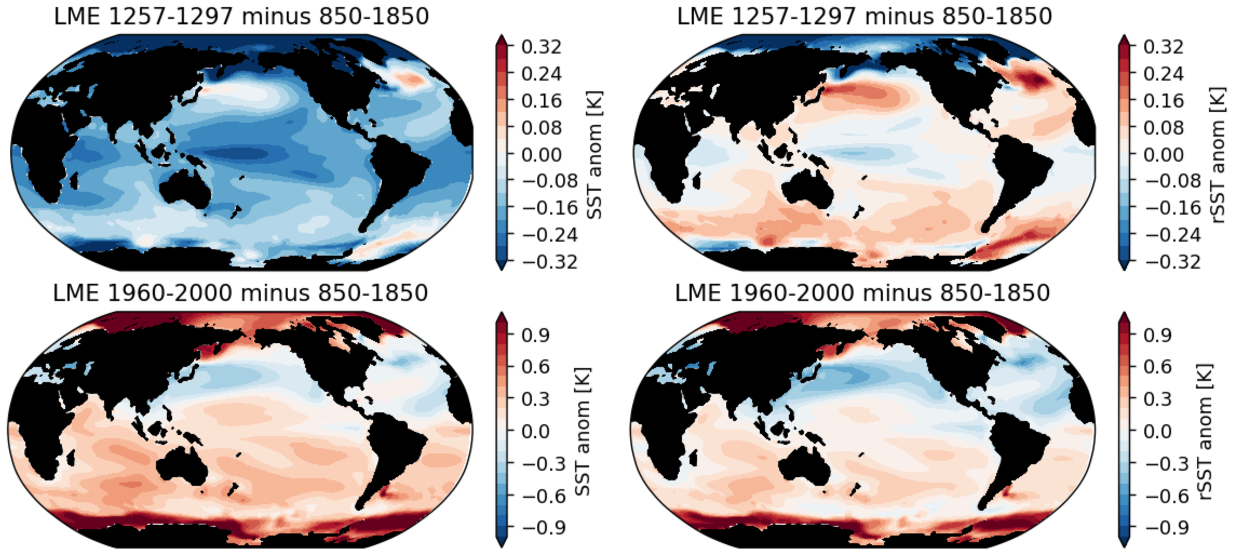

Supplementary Figure S5: SST (left column) and relative sea surface temperature (SST) (right column) anomalies over 1257-1297 and 1960-2000 compared to the reference period of 850-1850 from Last Millennium Ensemble (LME) full forcing experiment. Source data are provided as a Source Data file.

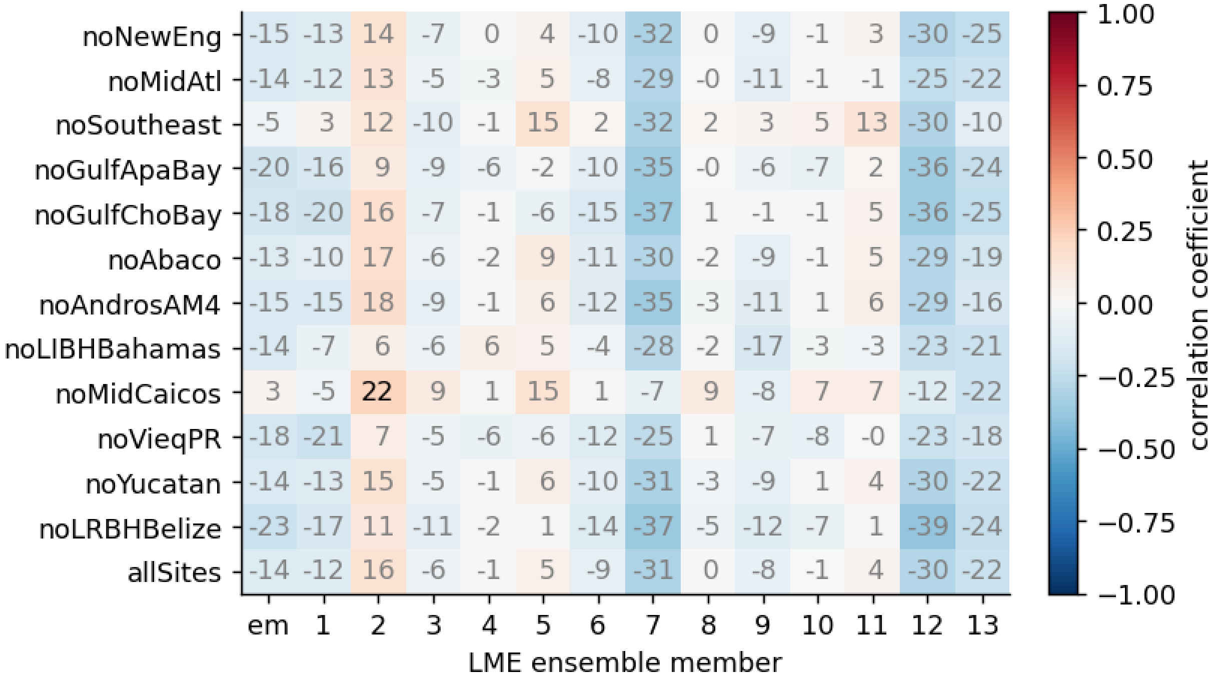

Supplementary Figure S6: Same as Supplementary Figure S1 except that hurricanes (HU) based on Last Millennium Ensemble (LME) sea surface temperatures (SSTs) from the full forcing experiment are analyzed. Source data are provided as a Source Data file.

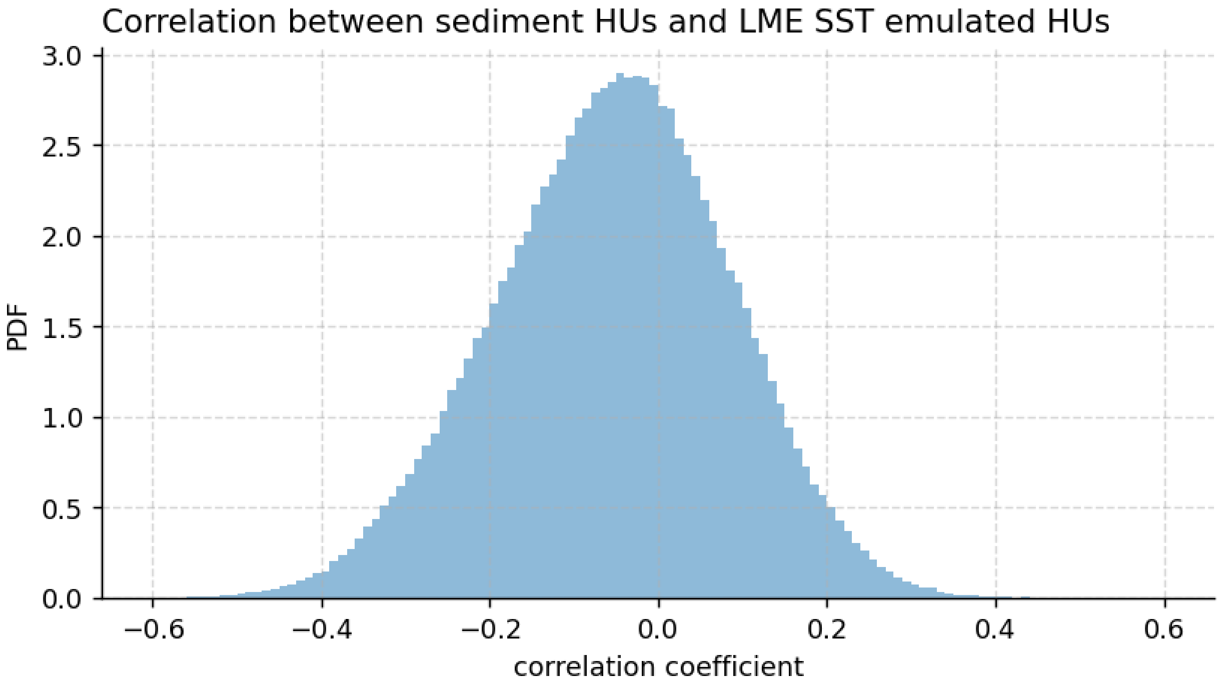

Supplementary Figure S7: Same as Supplementary Figure S2 except that hurricanes (HUs) based on Last Millennium Ensemble (LME) sea surface temperature (SSTs) from the full forcing experiment are analyzed. Source data are provided as a Source Data file.
